# Effects of a High Fat Diet and Voluntary Wheel Running Exercise on Cidea and Cidec Expression in Liver and Adipose Tissue of Mice

**DOI:** 10.1371/journal.pone.0130259

**Published:** 2015-07-15

**Authors:** Thomas H. Reynolds, Sayani Banerjee, Vishva Mitra Sharma, Jacob Donohue, Sandrine Couldwell, Alexandra Sosinsky, Ashton Frulla, Allegra Robinson, Vishwajeet Puri

**Affiliations:** 1 Department of Health and Exercise Sciences, Skidmore College, Saratoga Springs, NY, 12866, United States of America; 2 Department of Medicine, Section of Endocrinology, Diabetes and Nutrition, Boston University, Boston, MA, 02118, United States of America; John Hopkins University School of Medicine, UNITED STATES

## Abstract

Cidea and Cidec play an important role in regulating triglyceride storage in liver and adipose tissue. It is not known if the Cidea and Cidec genes respond to a high fat diet (HFD) or exercise training, two interventions that alter lipid storage. The purpose of the present study was to determine the effect of a HFD and voluntary wheel running (WR) on Cidea and Cidec mRNA and protein expression in adipose tissue and liver of mice. A HFD promoted a significant increase in Cidea and Cidec mRNA levels in adipose tissue and liver. The increase in Cidea and Cidec mRNAs in adipose tissue and liver in response to a HFD was prevented by WR. Similar to the changes in Cidea mRNA, Cidea protein levels in adipose tissue significantly increased in response to a HFD, a process that was, again, prevented by WR. However, in adipose tissue the changes in Cidec mRNA did not correspond to the changes in Cidec protein levels, as a HFD decreased Cidec protein abundance. Interestingly, in adipose tissue Cidea protein expression was significantly related to body weight (R=.725), epididymal adipose tissue (EWAT) mass (R=.475) and insulin resistance (R=.706), whereas Cidec protein expression was inversely related to body weight (R=-.787), EWAT mass (R=-.706), and insulin resistance (R=-.679). Similar to adipose tissue, Cidea protein expression in liver was significantly related to body weight (R=.660), EWAT mass (R=.468), and insulin resistance (R=.599); however, unlike adipose tissue, Cidec protein levels in liver were not related to body weight or EWAT mass and only moderately associated with insulin resistance (R=-.422, P=0.051). Overall, our findings indicate that Cidea is highly associated with adiposity and insulin resistance, whereas Cidec is related to insulin sensitivity. The present study suggests that Cide proteins might play an important functional role in the development of obesity, hepatic steatosis, as well as the pathogenesis of type 2 diabetes.

## Introduction

The most recent data from the CDC reveals that diabetes affects almost 26 million people in the United States [1], resulting in an estimated $245 billion in total health care costs [2]. Approximately 90–95% of diagnosed cases of diabetes are type 2 diabetes, a disease highly associated with insulin resistance and obesity. Even more alarming than the diabetes statistics is the prevalence of obesity. Data from the National Health and Nutrition Examination Survey demonstrates that 35.7% of United States adults are obese [3]. Because of the prevalence and devastating health consequences of obesity, it is important to gain a better understanding of factors that regulate fat storage in insulin sensitive tissues, particularly since lipids have been shown to disrupt insulin signaling and promote insulin resistance [4].

Excess dietary lipids are stored within adipocytes as lipid droplets, dynamic organelles whose surface is composed of hydrophilic phospholipids and regulatory proteins. The “PAT” protein family is a group of proteins that bind lipid droplets and regulate both the accumulation and mobilization of triglycerides within a lipid droplet [5]. Perilipin (Plin1) is the founding member of the perilipin/adipophilin/tail-interacting protein of 47 kDa (PAT) family. Plin1 contains several PKA phosphorylation sites and mediates the lipolytic effect of beta adrenergic signaling, whereas Plin2 appears to promote triglyceride accumulation [6]. Other PAT family members such as ADRP, S3-12, and OXPAT have important functional roles that dictate lipid droplet size and morphology [5]. Recently, Plin1 has been shown to promote lipid droplet growth through an interaction with fat specific protein 27 (Fsp27), a member of the cell death- inducing DFF45 effector (Cide) family of proteins [7]. The Cide family consists of Cidea, Cideb and Cidec (also called Fsp27), all of which have been shown to associate with lipid droplets. In fact, the lipid binding domain of Cidea and Cidec has been identified [8], and several reports indicate that Cide proteins play an important role in regulating lipid droplet size and triglyceride accumulation [9–11]. Similar to Plin knockout mice [12], genetic ablation of Cidea or Cidec results in a lean phenotype, enhanced insulin sensitivity, and resistance to dietary induced obesity [13, 14].

Although studies of knockout mice and *in vitro* molecular investigations clearly show that Cide proteins associate with lipid droplets and regulate metabolism, little *in vivo* information exists regarding Cide expression in response to interventions that alter adipose tissue mass and insulin action. We recently conducted microarray gene expression analysis in livers from diet-induced obese mice and observed a substantial increase in Cidea and Cidec expression. Therefore, the purpose of the present study was to determine the effects of diet-induced obesity and voluntary wheel running (WR) on Cidea and Cidec mRNA and protein expression in liver and adipose tissue. Our hypothesis was that Cidea and Cidec mRNA and protein expression would increase in response to a high fat diet (HFD) and decrease in response to voluntary WR. To test this hypothesis, mice were placed on either a low fat diet (LFD) or a HFD and housed in either standard cages or cages equipped with running wheels.

## Methods

### Animals

Wildtype C57B6 male mice were purchased from the Jackson Laboratory (Bar Harbor, ME). Upon arrival to the animal facility at Skidmore College, all mice (~1 month old) were housed individually with cage enrichment nest-lets and fed ad libitum chow and water for one week. Mice were then randomly assigned to either WR cages (Mini-Mitter, Bend, OR) or standard cages and placed on a HFD (Test Diets, Catalog #1810251,60% kcal from fat) or a LFD (Test Diets, Catalog #58145,12% kcal from fat) (Test Diets, St. Louis, MO). All animal care and surgery were conducted in accordance with the National Research Council's Guide for Care and Use of Laboratory Animals (Institute of Laboratory Animal Resources, Commission on Life Sciences, 1996). All experimental protocols were approved by Skidmore College's Institutional Animal Care and Use Committee.

### In vivo insulin action

To assess the effects of a HFD and voluntary WR on *in vivo* insulin action, mice were subjected to an insulin-assisted glucose tolerance (IAGT) test. Prior to the IAGT test mice were fasted overnight and remained in either standard or WR cages. Following the overnight fast, mice were simultaneously administered glucose (2 g/Kg body weight) and insulin (2 U/Kg body weight) by intraperitoneal injection. Glucose was measured by a glucometer in blood collected via the tail vein at 0, 20, 40, and 60 min following the glucose/insulin injection. We have previously shown that the IAGT test detects insulin resistance as well as an insulin tolerance test in mice fed a HFD, but avoids the severe hypoglycemia that is typically observed during and after insulin tolerance testing [15].

### Surgical procedures

Prior to harvesting tissues, mice were fasted overnight and remained in either standard or WR cages. Mice were then anesthetized with a 1:1:1 mixture of promace, ketamine hydrochloride, and xylazine by an intraperitoneal injection (0.015 ml/10 g body weight). Liver, epididymal white adipose tissue (EWAT), and soleus muscles were rapidly dissected, frozen in liquid nitrogen, and stored at –80°C until analysis.

### RNA extraction and real time quantitative PCR

Total RNA was extracted from Liver and EWAT using an RNA extraction kit for high lipid content tissue (Qiagen) and soleus muscle RNA was extracted using an RNA kit for fibrous tissues. RNA was quantified by measuring absorbance at 260 nm using a spectrophotometer (Beckman Coulter, Brea, CA). A 1 ug aliquot of total RNA was reverse transcribed using the RETROscript kit from Ambion (Austin, TX). The resultant cDNA (20 ng cDNA/sample in duplicate) was then subjected to quantitative polymerase chain reaction (qPCR) using standard target specific TaqMan gene expression assays for Cidea (Assay ID: Mm00432554_m1), Cidec (Assay ID: Mm00617672_m1), peroxisome proliferator activated receptor gamma (PPARγ) (Assay ID:), PPARγ coactivator-1 alpha (PGC-1α) (Assay ID: Mm01208835_m1) and a real time PCR system (StepOne Plus Real-Time PCR System, Applied Biosystems, Foster City, CA). Relative quantitation of amplified cDNA targets were determined by the ΔΔCT method using StepOne v2.1 software (Applied Biosystems).

### Preparation of tissue extracts and electrophoretic analyses and immunoblotting

Frozen liver and EWAT were homogenized on ice in RIPA Buffer (Sigma Chemical, Inc.) (10:1 buffer to QUAD mass and 5:1 buffer to EWAT mass ratios) containing protease and phosphatase inhibitor cocktails (Halt Protease/Halt Phosphatase, Thermo Fisher). Homogenates were rotated at 4°C for 1 h and centrifuged at 9,000 x g for 30 min at 4°C. The protein concentrations of the supernatants were determined by the BCA method (Pierce, Inc), and equal amounts of protein were subjected to SDS-PAGE along with molecular weight standards (Bio-Rad, Hercules, CA and Magic Mark, Invitrogen). Proteins were then electrophoretically transferred to Immobilon membranes and immunoblotted with Cidea and Cidec antibodies. Light generated by the alkaline phosphatase conjugated secondary antibody and CDP-Star reagent was detected using a digital imaging system (UVP, Upland, CA). To account for gel loading differences, all immunoblots were stripped and re-probed with a ß-tubulin antibody. Relative signal intensities of immunoreactive bands were determined using Total Lab software (Nonlinear, Inc., Durham, NC) or GraphPad Prism and ImageJ. All data were normalized to ß-tubulin and expressed as a percentage of LFD-Vehicle. The ß-actin antibody was from Cell Signaling, the Cidea antibody was from Sigma, and the Cidec antibody was a kind gift from Cynthia Smas, Toledo, Ohio.

### Statistical analysis

To detect statistical significance for all dependent variables, a two-way analysis of variance (ANOVA) was utilized to detect the main effects for diet (LFD vs. HFD) and exercise (SED vs. WR). Following a significant F ratio and inspection of interactions, Fisher’s LSD post-hoc test was used to locate statistically significant differences between groups. The strength of relationships between Cide protein expression and Body mass, EWAT mass, and IAGTT-AUC were determined by Pearson product-moment correlation coefficient (R-value). Data are expressed as means ± SEM, and the level of statistical significance was set at p ≤ 0.05. For mRNA expression, means ± SEM were expressed relative to the LFD-SED group mean (% LFD-SED).

## Results

### Cidea and Cidec mRNA and protein expression

**Fig 1:**
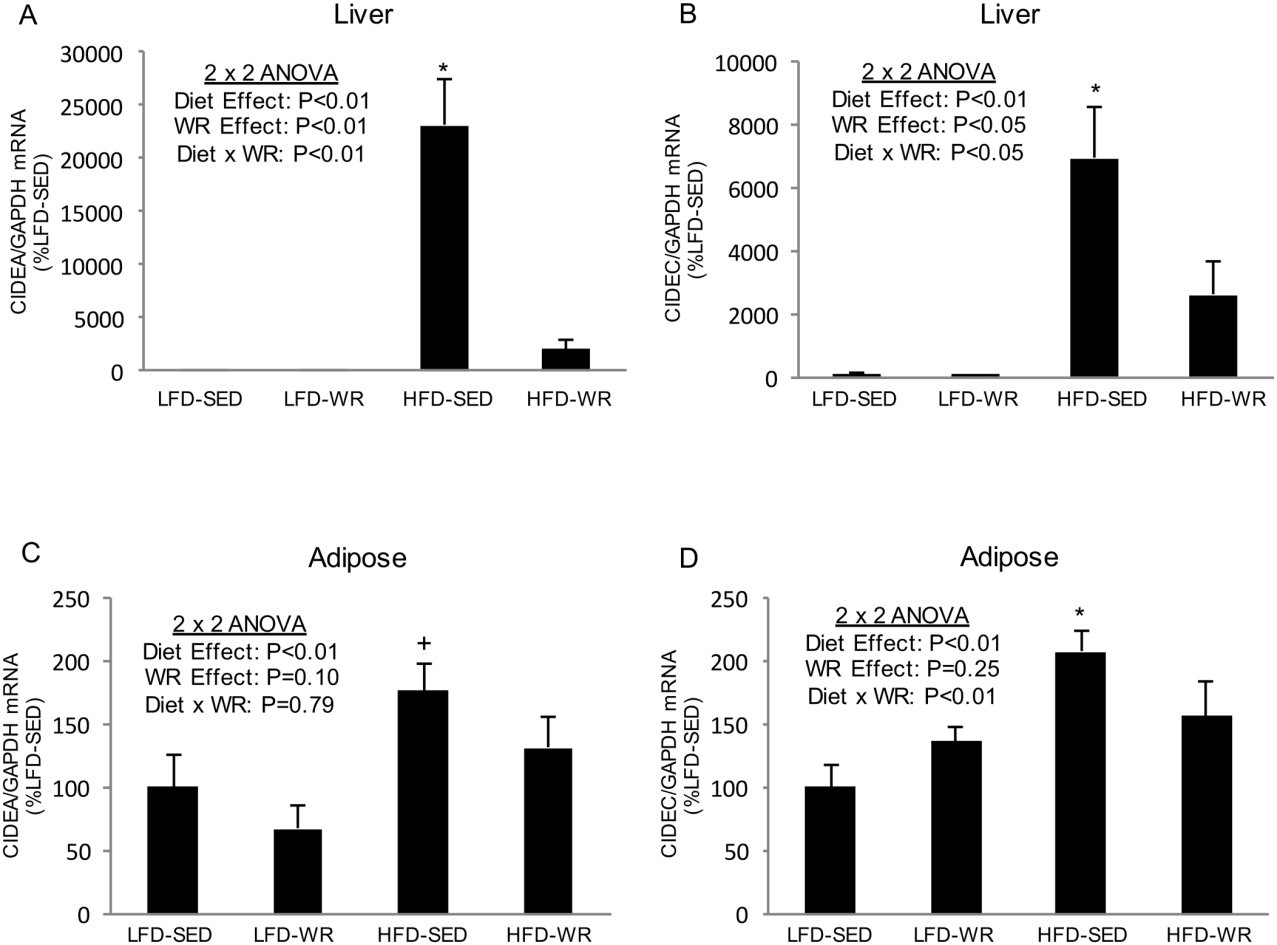
The effects of a high fat diet and voluntary wheel running on Cidea and Cidec mRNA expression in liver and adipose tissue. Cidea and Cidec gene expression for liver (Panels A and B) and epididymal white adipose tissue (Panels C and D) were normalized to GAPDH and expressed relative to LFD-SED values. *Denotes statistically significant difference from all other groups by Fisher’s LSD post-hoc test. ^+^Denotes statistically significant difference from LFD-SED and LFD-WR groups by Fisher’s LSD post-hoc test. The number of mice per group is 4–5.

**Fig 2:**
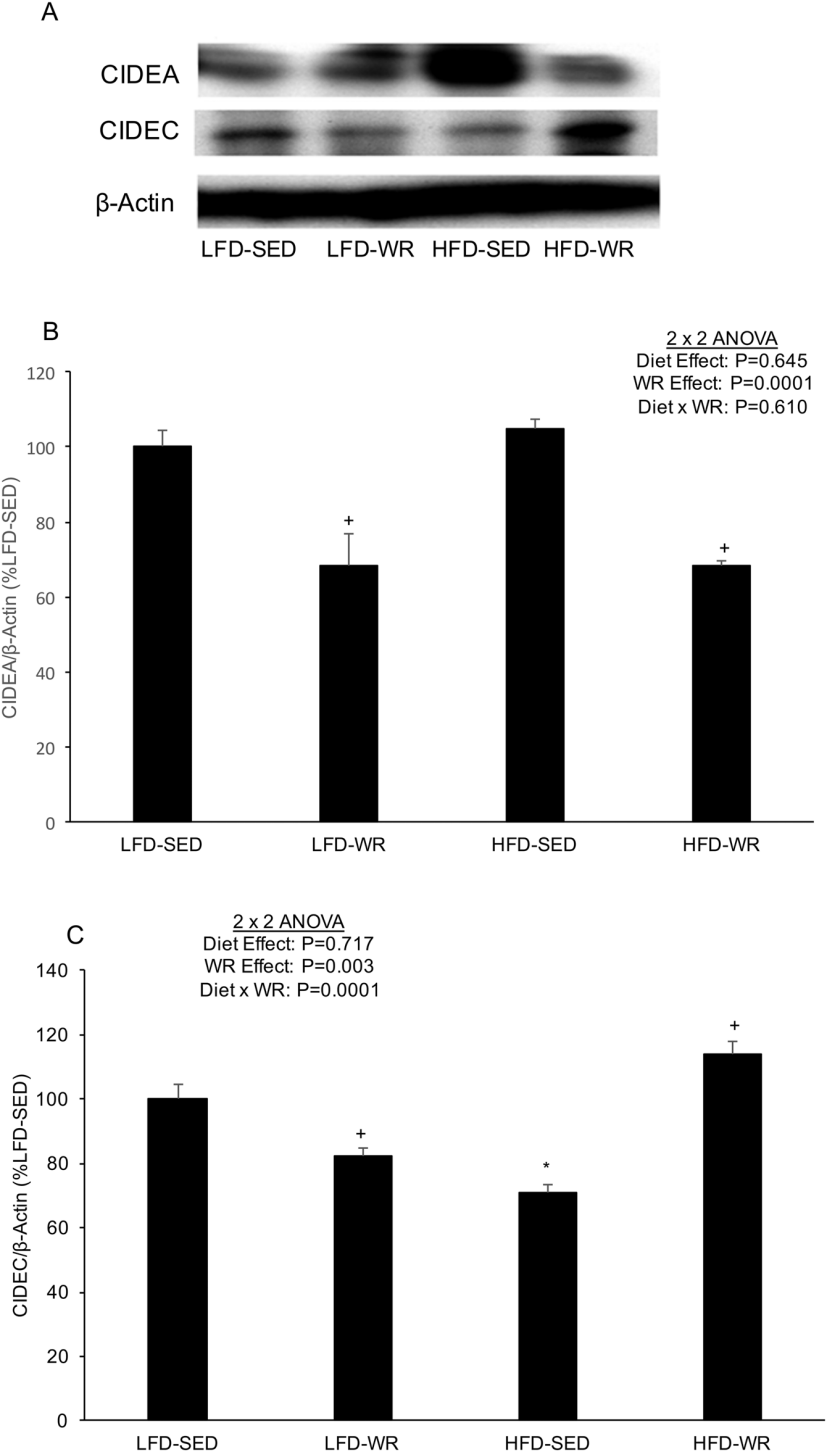
The effects of a high fat diet and voluntary wheel running on Cidea and Cidec protein expression in liver. Mice were fed a LFD or HFD and housed in standard cages or wheel running cages for weeks. Liver was excised and rapidly frozen in liquid nitrogen for subsequent Western blot analyses. **(A)** Representative immunoblots for Cidea and Cidec as well as β-actin as a loading control. Quantification of **(B)** Cidea and **(C)** Cidec protein, each normalized to β-actin. *, Denotes statistically significant difference from HFD-Vehicle. N = 5 mice per group.

**Fig 3:**
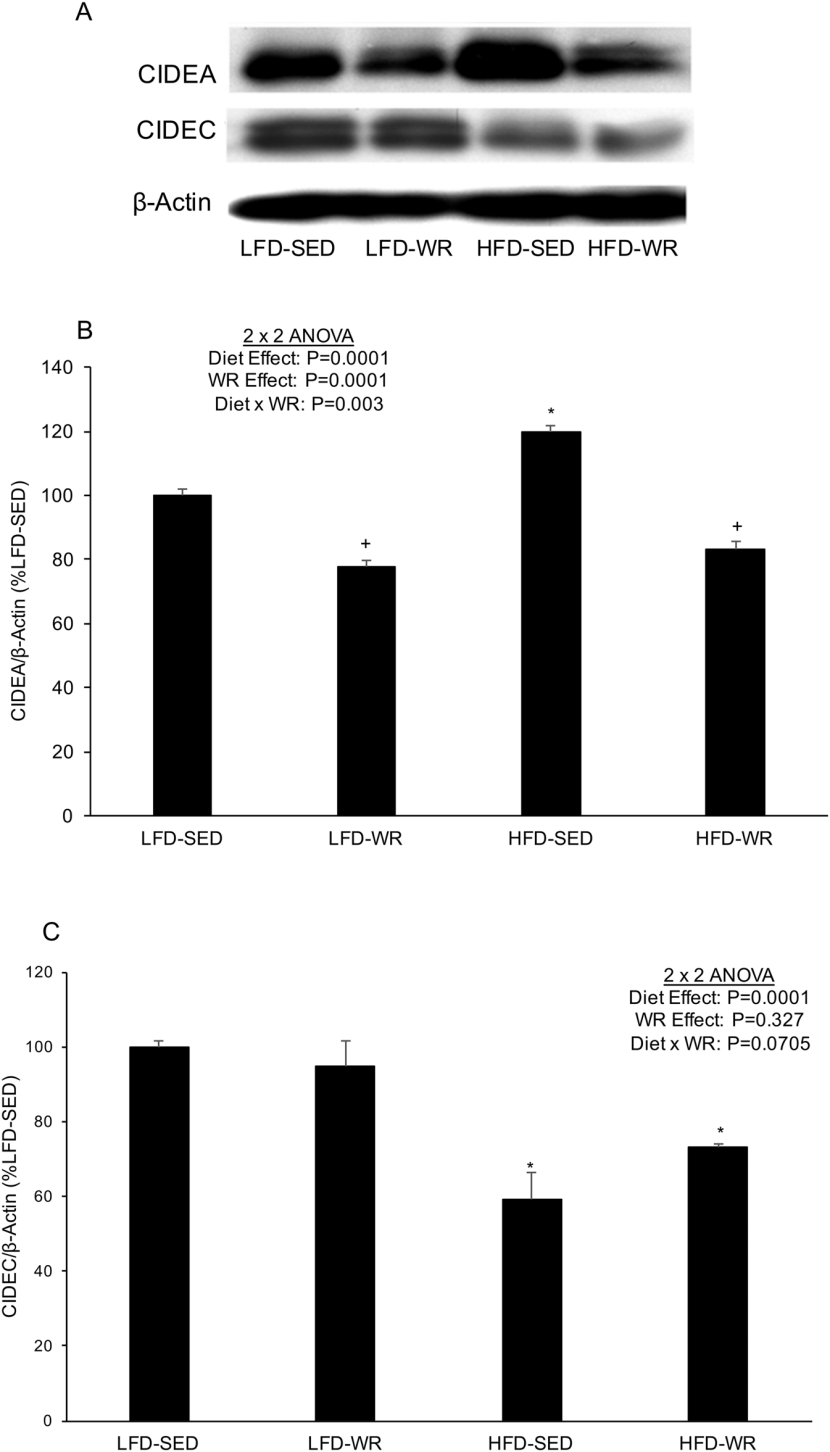
The effects of a high fat diet and voluntary wheel running on Cidea and Cidec protein expression in adipose tissue. Mice were fed a LFD or HFD and housed in standard cages or wheel running cages for weeks. Adipose tissue was excised and rapidly frozen in liquid nitrogen for subsequent Western blot analyses. **(A)** Representative immunoblots for Cidea and Cidec as well as β-actin as a loading control. Quantification of **(B)** Cidea and **(C)** Cidec protein, each normalized to β-actin. *, Denotes statistically significant difference from HFD-Vehicle. N = 5 mice per group.

Since previous studies have shown the role of Cidea and Cidec in triglyceride accumulation in adipocytes [16, 17], the effect of a HFD and WR was assessed on the expression of these genes in liver and adipose tissues. As shown in Fig 1A and 1B, Cidea and Cidec expression were significantly higher in livers from mice fed a HFD compared to mice fed a LFD (> 200- and 60-fold increase, respectively). The dramatic increase in hepatic Cidea and Cidec expression due to diet-induced obesity appears to be abolished by WR (Fig 1A and 1B). The expression of Cidea and Cidec protein in liver did not correspond with the mRNA expression as a HFD produced no changes or a decline in Cidea and Cidec abundance, respectively (Fig 2). In response to WR Cidea protein levels decreased in livers from mice fed a LFD and HFD. Although Cidec protein levels increased in response to WR in HFD-fed mice, WR produced a significant decrease in Cidec levels in LFD-fed mice. In adipose tissue, Cidea mRNA expression was significantly higher in SED mice fed a HFD compared to SED mice fed a LFD (Fig 1C and 1D), although this effect was not nearly as robust as in liver (~1.75-fold increase vs. > 200-fold increase). Cidea mRNA expression in adipose tissue was not significantly altered by WR in mice fed either a HFD or LFD. Similar to Cidea expression, Cidec expression was significantly higher in adipose tissue from mice fed a HFD compared to a LFD; however, this effect appears to be prevented by voluntary WR as Cidec expression in adipose tissue from HFD-WR mice was significantly lower than HFD-SED mice (Fig 1D). The expression of Cidea protein in adipose tissue corresponded nicely with the mRNA expression as a HFD produced an increase and WR produced a decrease in Cidea protein abundance (Fig 3A and 3B). However, Cidec protein expression did not correspond with Cidec mRNA expression as a HFD produced a decrease, rather than an increase, in Cidec protein abundance (Fig 3A and 3C). Similar to Cidec mRNA levels, WR did not alter Cidec protein expression in adipose tissue of either the LFD- or HFD-fed mice.

### PPARγ and PGC-1α mRNA expression

**Fig 4:**
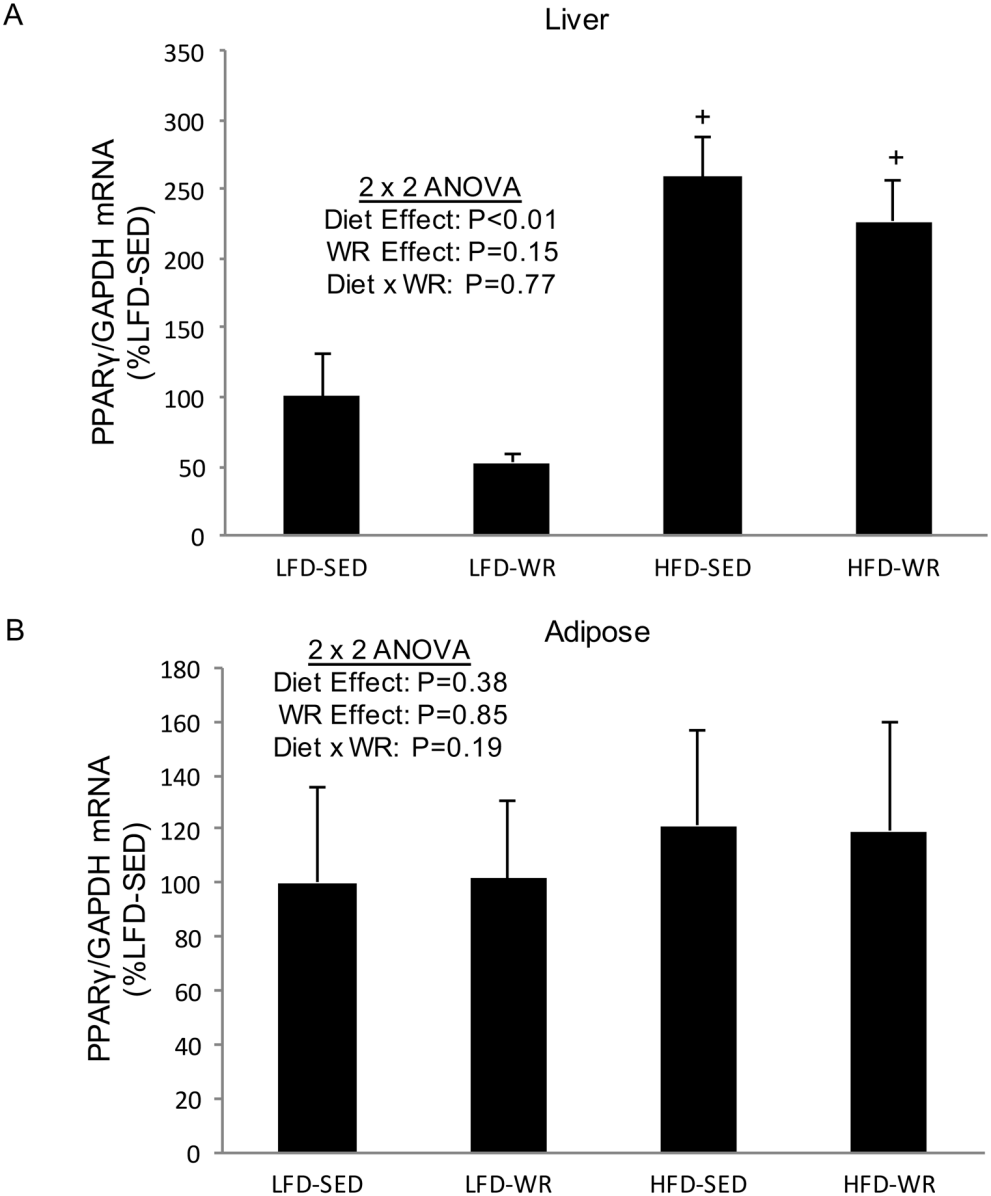
The effects of a high fat diet and voluntary wheel running on PPARγ mRNA expression in liver and adipose tissue. PPARγ gene expression for liver (Panel A) and epididymal white adipose tissue (Panel B), were normalized to GAPDH and expressed relative to LFD-SED values. ^+^Denotes statistically significant difference from LFD-SED and LFD-WR groups by Fisher’s LSD post-hoc test. The number of mice per group is 5.

PPARγ is a transcription factor which regulates adipocyte differentiation, lipid metabolism, and insulin sensitivity, and has been shown to play a role in the regulation of Cidea and Cidec expression in adipocytes [16, 18]. Therefore, we wanted to study its expression in liver and adipose tissue of mice subjected to a HFD and/or WR. We found that PPARγ expression is increased significantly in livers of mice fed a HFD compared to a LFD (Fig 4A). However, in adipose tissue and skeletal muscle PPARγ was unaltered by a HFD (Fig 4B). In addition, voluntary WR did not change PPARγ expression in any tissue studied (Fig 4A and 4B).

**Fig 5:**
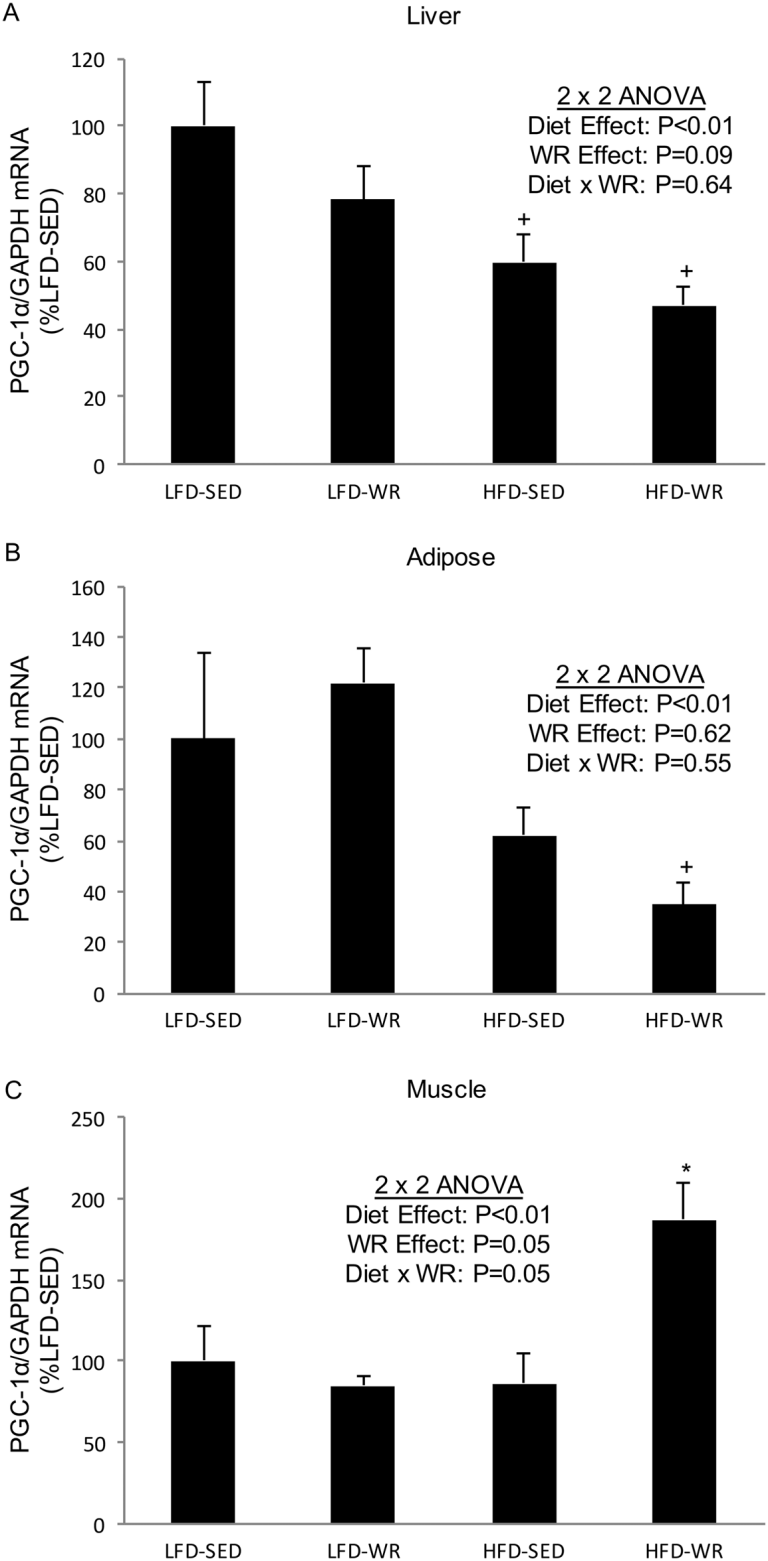
The effects of a high fat diet and voluntary wheel running on PGC-1α mRNA expression in liver, adipose tissue, skeletal muscle. PGC-1α gene expression for liver (Panel A), epididymal adipose tissue (Panel B), and soleus muscle (Panel C) were normalized to GAPDH and expressed relative to LFD-SED values. *Denotes statistically significant difference from all other groups by Fisher’s LSD post-hoc test. ^+^Denotes statistically significant difference from respective LFD groups by Fisher’s LSD post-hoc test. The number of mice per group is 4–5.

The PPARγ coactivator, PGC-1α, is a well-established regulator of mitochondrial biogenesis whose expression increases in skeletal muscle following exercise training [19, 20]. Furthermore, PGC-1α has been shown to participate in the regulation of Cidea [21]. Here we show that PGC-1α expression increases in soleus muscles from mice fed a HFD and given free access to a running wheel (Fig 5C); however, PGC-1α levels were not altered by voluntary WR in muscles from mice fed a LFD. In liver and adipose tissue, PGC-1α expression is unaltered by voluntary WR but decreases in response to a HFD (Fig 5A and 5B), despite the significant increase in Cidea and Cidec expression.

### Adiposity

To assess the effects of a HFD and WR on adiposity, we measured body weight and EWAT mass (Table 1). As expected, 10 weeks of a HFD produced significant increases in body weight and EWAT mass in sedentary mice that did not have access to a running wheel. In mice fed a LFD and housed in cages equipped with running wheels, there were significant reductions in body weight and EWAT mass compared to LFD mice housed in standard cages. In mice fed a HFD and housed in cages equipped with running wheels, there was a significant reduction in body weight, but EWAT mass was similar to HFD mice housed in standard cages. Overall, a significant positive correlation existed between adiposity (body weight, EWAT mass) Cidea protein levels in liver and adipose tissue (Table 2). However, a significant inverse relationship existed between adiposity and Cidec protein expression only in adipose tissue (Table 2).

**Table 1:** The effects of a high fat diet and voluntary wheel running on body mass, epididymal white adipose tissue (EWAT) mass, and caloric intake.

	Fasted Body Mass(g)	Fed Body Mass (g)	EWAT Mass (g)	Caloric Intake (kcal/day)
CON-SED (n = 8)	29.4±0.9	32.4±0.8	1.20±0.11	16.78±0.47
CON-WR (n = 5)	23.7±0.5^+^	27.8±0.5^+^	0.55±0.03^+^	17.16±0.51
HFD-SED (n = 9)	40.3±0.7*	44.1±0.8*	2.01±0.10*	20.18±0.32*
HFD-WR (n = 6)	32.5±2.0^+^	36.6±1.9^+^	1.79±0.19	20.58±0.51

^+^Significantly different from SED group on respective diet by Fisher’s LSD post-hoc test

*Significantly different from CON-SED group by Fisher’s LSD post-hoc test

**Table 2:** CIDE protein expression in adipose tissue and liver from CON-SED, CON-WR, HFD-SED, and HFD-WR mice is related to measures of adiposity and insulin resistance.

	Body Mass Correlation Coefficient (R)	EWAT Mass Correlation Coefficient (R)	IAGTT (AUC) Correlation Coefficient (R)
Adipose Tissue			
CIDEA	0.725*	0.475*	0.706*
CIDEC	-0.787*	-0.706*	-0.679*
Liver			
CIDEA	0.660*	0.468*	0.599*
CIDEC	-0.212*	0.096*	0.442*

*Significant Pearson product-moment correlation coefficient, P value is ≤.05.

Tissues from 20 mice were used for the analysis.

### In vivo insulin action

**Fig 6:**
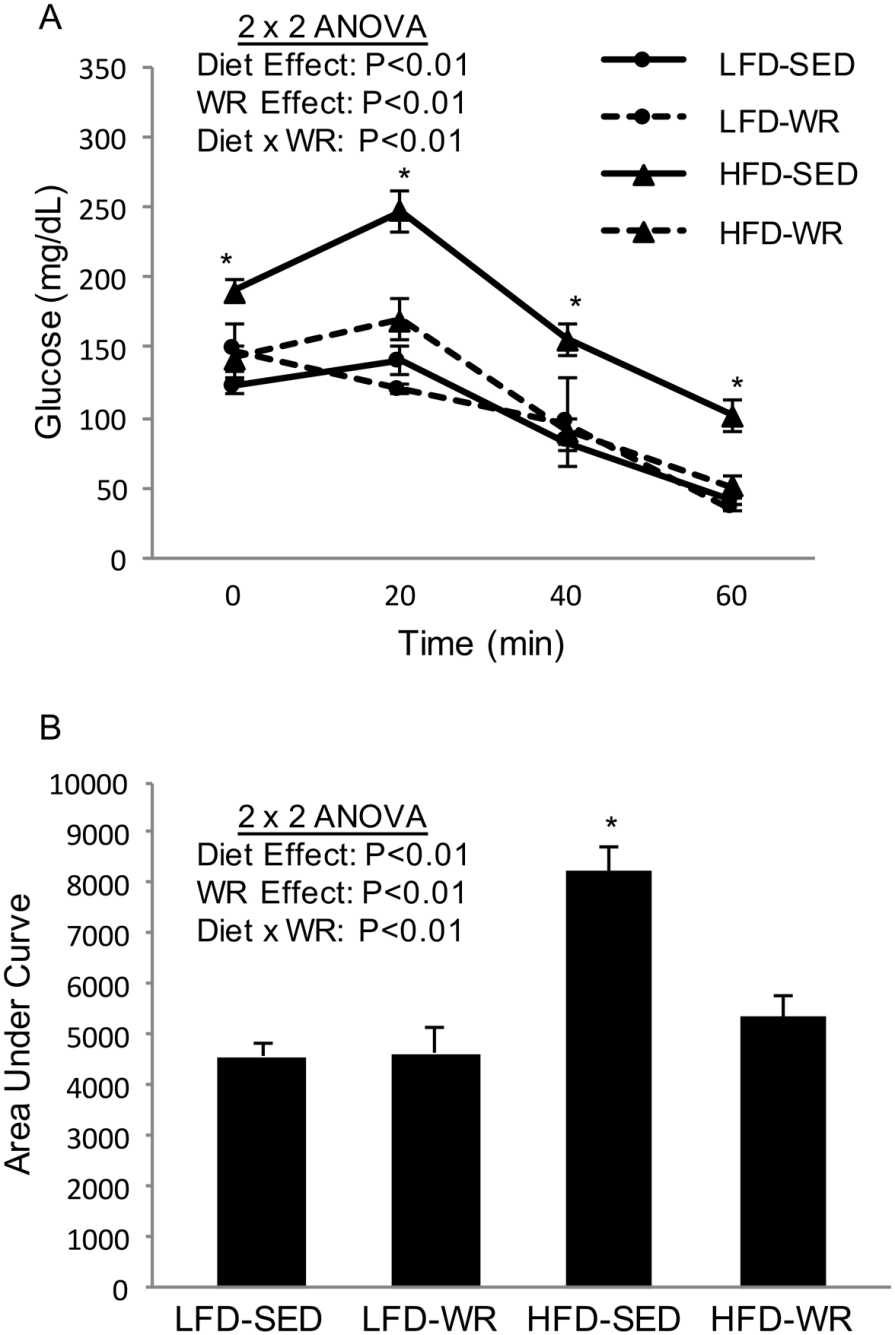
The effects of a high fat diet and voluntary wheel running on insulin-assisted glucose tolerance. Mice received a simultaneous injection of glucose (2 mg/Kg) and insulin (2 U/Kg), and glucose was measured in blood collected from the tail vein at 0, 20, 40, and 60 minutes following the injection (Panel A). The area under the insulin-assisted glucose tolerance curve (AUC) was calculated from blood glucose values during the IAGTT (Panel B).*Denotes statistically significant difference from all other groups by Fisher’s LSD post-hoc test. The number of mice per group is 6–9.

*In vivo* insulin action was determined by conducting IAGT testing, a procedure that has been to detect diet-induced insulin resistance at more physiological relevant blood glucose levels than insulin tolerance testing [15, 22]. In the fasted basal state, blood glucose values were higher in the HFD-SED mice compared to all other groups (HFD-SED: 190.1 ± 8.4 vs. HFD-WR: 142 ± 9.1 vs. LFD-SED: 122.1 ± 4.6 vs. LFD-WR: 147.9 ± 18.8). Blood glucose levels of HFD-SED mice were significantly higher than LFD-SED mice at 20, 40, and 60 min following a simultaneous injection of glucose and insulin, indicating the presence of diet-induced insulin resistance (Fig 6A). Diet-induced insulin resistance was completely prevented by WR as blood glucose values for HFD-WR mice were similar to LFD-SED mice (Fig 6A). Fig 6B shows that the area under the curve (AUC) is significantly higher in HFD-SED mice compared to LFD-SED, a process that was almost completely prevented by WR. Unlike HFD mice, mice fed a LFD given free access to a running wheel did not experience a reduction in blood glucose levels during the IAGT test. The changes in the AUC due to a HFD or WR were high related to Cidea protein expression in liver and adipose tissue; however, Cidec protein expression was inversely related to the AUC (Table 2). It is important to note that the reduction in body weight in the HFD-WR group likely contributed to in the improvement in insulin action.

## Discussion

Although the importance of Cide expression in the regulation of triglyceride storage and metabolism has been established in Cide knockout mice and in *in vitro* experiments [13, 14, 23], to the best of our knowledge, this is the first report that demonstrates the effect of a HFD and/or exercise training on Cidea and Cidec mRNA and protein expression in adipose tissue from wild type mice. The most novel aspect of our study is the observation that exercise training greatly reduces the ability of a HFD to increase Cidea protein expression in adipose tissue and that Cidea expression is tightly correlated with adiposity and insulin resistance. Although a HFD did not alter hepatic Cidea protein expression, wheel running exercise reduced Cidea protein levels. In contrast to the increase in adipose tissue Cidea protein abundance following a HFD, Cidec protein expression decreases in adipose tissue and is inversely related to adiposity and insulin resistance. Furthermore, hepatic Cidec levels decrease in response to a HFD and are inversely related to insulin resistance. Although these results are limited by not assessing tissue triglyceride levels, the present study provides an initial observation that suggests Cides might play an important functional role in the regulation of adiposity and insulin action.

The present study demonstrates that feeding mice a HFD increases Cidea mRNA and protein expression in adipose tissue, an observation that appears to be related to adiposity and insulin resistance. In fact, we show significant relationships between Cidea protein levels and body weight (R = .725), adipose tissue mass (R = .475), and IAGT test AUC (R = .706). In addition, the relationship between hepatic Cidea protein expression is strongly and significantly correlated with adiposity and insulin resistance. In humans, adipose tissue Cidea expression is related to insulin sensitivity [16, 17], an observation that is distinct from the present findings and that of the lean, insulin sensitive phenotype of Cidea knockout mice [13]. The higher Cidea expression in adipose tissue from insulin sensitive humans might allow for greater triglyceride storage and may protect other insulin sensitive tissue such as skeletal muscle, thereby preserving insulin responsiveness [16, 24]. Although the present study observed a significant increase in Cidea expression in adipose tissue and a decreased whole body insulin sensitivity, perhaps if the increase in Cidea levels in adipose tissue in response to a HFD was more robust, insulin action may not have deteriorated. In humans, the observation that PPARγ agonists increase adipose tissue Cidea expression and facilitate greater lipid accumulation [16] supports the notion that in adipose tissue Cidea can preserve insulin action by appropriately storing triglycerides and preventing lipotoxicity in non-adipose tissue. Likewise, a greater increase in hepatic Cidea protein expression in response to a HFD may be protective against steatosis.

The increase in adipose tissue Cidea protein abundance is likely a compensatory response for the decrease in Cidec protein. Surprisingly, Cidec protein expression decreased in both adipose tissue and liver from mice fed a HFD. Overall, robust inverse relationships existed between adipose tissue Cidec protein expression with body weight (R = -.787), EWAT mass (R = -.706), and insulin resistance (R = -.679). In liver, Cidec was inversely related to insulin resistance (R = -.442). Recently, Cidec has been shown to play an important role in regulating lipolysis by interacting with ATGL and preventing its lipolytic function [23], and also affecting ATGL transcription [25]. Therefore, our finding that a HFD reduces Cidec protein expression indicates that lipolysis is unrestrained and contributing to fatty acid induced insulin resistance [4]. Since Cidea expression is regulated by fatty acids [26], the loss of Cidec protein expression may have initiated an increase in Cidea expression indirectly by increased lipolysis.

Arguing against the idea that adipose tissue Cidea protein expression preserves insulin sensitivity, the present study demonstrates that voluntary WR prevents an increase in adipose tissue and hepatic Cidea levels but improves insulin action in mice. The reduction in Cidea in both adipose tissue and liver from mice given free access to a running wheel is likely due to a decrease in lipid availability, a process that is likely mediated by enhanced lipid utilization in exercising skeletal muscle [27, 28]. Interestingly, the effect of exercise on Cidec protein expression is quite different from Cidea, as 10 weeks of voluntary WR increases Cidec protein expression in adipose tissue and liver, at least in HFD-fed mice. The increase, or at least the attenuation of a decline, in Cidec protein levels following WR could be related to an exercise-induced activation of the sympathetic nervous system as the β-adrenergic receptor agonist isoproterenol or cAMP analogs have been shown to increase Cidec expression [29].

The molecular events responsible for the changes in Cidea and Cidec expression in liver and adipose tissue in response to diet and exercise interventions are not well-established. With the exception of Cidea expression in adipose tissue, the present study observed quite different Cide mRNA responses to diet and exercise compared to the protein, indicating post-transcriptional regulation. In fact, the dramatic increase in hepatic Cidec mRNA in response to a HFD appears to be a futile attempt to off-set the reduction in Cidec protein levels (Fig 1B and Fig 2C). At least in adipocytes, Cidec protein abundance is regulated by the ubiquitin-proteasome system [30, 31].

The present study demonstrates that a HFD increases the hepatic expression of PPARγ (Fig 4A), a finding that likely plays an important role in the present increase in hepatic Cidea and Cidec mRNA levels. The increase in PPARγ is not surprising since the Cidea and Cidec promoters contain a putative peroxisome proliferator response element [18, 32], and the PPARγ agonist, rosiglitazone, has been shown to increase Cidea expression [16]. In addition to a HFD, voluntary WR appears to mediate a reduction in hepatic Cide mRNA expression. The reduction in Cidea and Cidec mRNA expression with voluntary WR occurs without a corresponding reduction in PPARγ, indicating that exercise training may reduce Cide expression independent of PPARγ, at least when consuming a HFD. Perhaps the persistent increase in PPARγ mRNA and the decrease in Cide mRNA is due to differences in the type of free fatty acids available to the livers from the HFD-WR group compared to the HFD-SED group, particularly since both Cidea and PPARγ expression is regulated by free fatty acids [26]. In other words, free fatty acids from saturated dietary fat likely promote Cide mRNA expression; whereas free fatty acids liberated from exercise may prevent this effect. It is important to note that other transcription factors such as SREBP1c and PPARα may play an important role in regulating the Cide mRNA expression [33–35]. Finally, one limitation to the present study is that we did not assess the total abundance or profile of free fatty acids following either a HFD or voluntary WR.

The transcriptional co-activator PGC-1α is a master regulator of mitochondrial biogenesis [36] that has recently been shown to regulate the expression of Cidea [21]. The present study showed a decrease in PGC-1α mRNA in liver and adipose tissue in response to a HFD, an observation that was directly opposite the large increase in Cide mRNA. In skeletal muscle, both exercise training and a HFD are associated with increased PGC-1α expression [19, 37, 37, 38], a finding that is not established in liver or adipose tissue. We observed no effect of a HFD on soleus muscle PGC-1α mRNA, but a significant increase in response to voluntary WR in mice fed a HFD. In the liver, PGC-1α plays an important role in promoting the expression of genes involved with gluconeogenesis during fasting [39]. Despite the need for gluconeogenesis to maintain blood glucose levels during exercise, the present study as well as others [40] show that voluntary WR does not increase PGC-1α expression in liver. In fact, exercise training may reduce PGC-1α expression in liver from obese mice [41]. In adipose tissue, obesity and insulin resistance are associated with lower levels of PGC-1α [42, 43], whereas increased expression of PGC-1α in adipose tissue can promote an insulin sensitive, lean phenotype [44, 45]. Likewise, exercise training via forced swimming of rats produced an increase in PGC-1α in visceral adipose tissue [46], however, we observed no significant increase PGC-1α levels in EWAT with voluntary WR. It is likely that voluntary WR is not a strong enough stimulus to induce PGC-1α expression, and forced swimming triggers a stress response (norepinephrine, epinephrine, thyroid hormone) resulting in PGC-1α expression.

In summary, we demonstrate significant changes in Cidea and Cidec mRNA and protein expression following a HFD and voluntary WR in liver and adipose tissue from mice. The most interesting aspect of the present study is the finding that exercise training greatly reduces the ability of a HFD to increase Cidea protein expression in adipose tissue and that Cidea expression is tightly correlated with adiposity and insulin resistance. In contrast, Cidec protein expression decreases in response to a HFD in both adipose tissue and is inversely related to adiposity and insulin resistance. Furthermore, hepatic Cidec levels decrease in response to a HFD and are inversely related to insulin resistance. Although these results are limited by not assessing tissue triglyceride levels, the present study suggests that Cidea and Cidec proteins might play an important functional role in the development of obesity, hepatic steatosis, as well as the pathogenesis of type 2 diabetes.
